# New Technique in Assessment of Heart Chambers Remodeling in Acquired Mitral Valve Defects

**DOI:** 10.3390/jcdd7020014

**Published:** 2020-04-21

**Authors:** Leo Bokeria, Vladimir Makarenko, Tatiana Kosareva

**Affiliations:** A.N. Bakulev National Medical Research Center of Cardiovascular Surgery, Moscow 121552, Russia; info@bakulev.ru (L.B.); vnmakarenko@heart-house.ru (V.M.)

**Keywords:** mitral valve disease, pathological remodeling, index

## Abstract

Objective: Analysis and presentation of the capabilities of the new ultrasound technique —the index of volume remodeling (IRV), which allows comprehensive assessing of pathological remodeling of the heart as an integrated functional anatomical system. Materials and methods: For this study 316 patients with acquired mitral valve disease (MVD) were examined prior to and following mitral valve replacement with bileaflet, disc-, and bioprostheses. Key parameters of the heart were measured in classical echocardiographic projections (end systolic area, end-diastolic area, end systolic volume, and end diastolic volume of ventricles, ventricular ejection fraction, atrial volume, and the ratio of ventricular to atrial volumes). The patients were examined 1–2 days prior to and following the surgery—before discharge, 6 months later, 1 year later, and then annually within next 5 years. The examination data were collected in one- and two-dimensional modes by using Philips EpiQ-7, iE33, HDI, Siemens Acuson, and HP Sonos 2500 diagnostic ultrasound machines equipped with 2.5 and 3.5 MHz transthoracic sensors. Results: A comprehensive study of structural geometric remodeling parameters of heart cavities in the context of acquired MVD allowed identifying new patterns in changes of the heart chambers geometry. These changes are reflected in the IRV, a digital indicator of the severity of cardiac pathological remodeling. Analysis of the dynamics of post-operative vs. pre-operative IRV-based remodeling data also showed that the index is highly sensible to the hemodynamic features of through-flows in various designs of prostheses. The IRV has a pronounced prognostic power and allows predicting the long-term outcome of surgical treatment with an accuracy of 82.35%. Conclusions: The IRV predictive accuracy formed the basis of the original classification of types of cardiac remodeling, which can assist both in determining the optimal timing for surgery, and in conjunction with other clinical diagnostic data, in predicting the long-term outcome of heart geometry restoration depending on the type of surgical correction. The IRV can be used in evaluation of the heart geometry for any cardiac pathology. It makes the approach to the analysis of pathological remodeling of the heart understandable, consistent, and universal, and also opens up opportunities for further expanding the diagnostic capabilities of radiology in cardiac surgery at all stages of the diagnostic process.

## 1. Introduction

Acquired heart defects represent a significant social problem as they affect people of socially active age and can lead not only to permanent disability, but also to death of patients, especially in case of ill-timed and inadequate treatment.

While conservative treatment methods have clear clinical performance criteria, the timing of surgical correction has no fully defined criteria and is left to the discretion of the treating cardiologist or a patient’s decision [1,2,3].

Presently the flags for determining the timing of surgical intervention are heart rhythm disturbances and clinical manifestations, which do not always reflect the severity of intracardiac hemodynamic impairment. Often patients do not feel rhythm disturbances or do not pay due attention to them. Arrhythmia in such case is diagnosed accidentally. Often patients notice heart failure symptoms only when they begin to significantly limit their social activity. 

The need for surgery is often associated with a noticeable decrease in the effectiveness of drug therapy and negative dynamics of a patient’s clinical condition. At this stage of the disease, impaired intracardiac hemodynamics often leads to resistant or almost irreversible pathological remodeling of the cardiac chambers. The surgical correction performed at this stage improves the heart geometry and the patient’s state of health, but the clinical manifestations of the disease and intracardiac hemodynamics impairment persist. They continue to disturb the patient and do not allow him to fully return to his social activity. Results of surgical correction at this stage of the heart disease development do not always meet patient’s expectations in terms of improved well-being.

Among surgical methods of treating MVD prosthetics remains the most widespread and frequently used type of surgical correction, despite great success in the development of valve-sparing reconstructive techniques. The timeliness of surgical correction determines the long-term results of treatment. The high technology surgery can reduce mortality by 3.5–5.0% during mitral valve replacement and in the early post-operative stages [4,5,6,7].

Postoperative patient survival largely depends on the initial state of intracardiac hemodynamics and severity of heart geometry changes (or pathological remodeling) that occurs in response to the damaging effect of hemodynamic impairment.

## 2. Materials and Methods

316 patients with acquired MVD were examined between 2004 to 2017 at the Bakulev National Medical Research Center of Cardiovascular Surgery (Moscow, Russia). Ethical Code at the A.N. Bakulev Research Centre: 19 October 2012, Directive No. 470 On Establishment of Local Ethical Committee. The selection criteria were the presence of anatomical and hemodynamic signs of acquired organic mitral valve pathology. A concomitant aortic valve defect was a factor of exclusion. The MVD was diagnosed based on the clinical picture of the disease, history, and results of clinical and instrumental examination. The average age of the patients was 50.3 + 18.5 years. 178 (66.6%) patients were female (average age- 57.4 + 12.8 years), 138 (33.4%) patients were male (average age- 51.6 + 15.8 years). The research sought to study changes in the heart chambers geometry caused by initial pathology of mitral valve and hemodynamic characteristics of the mitral prosthesis. The study focused on assessing the predictive power of the new ultrasound technique for measuring heart geometry changes in patients with MVD.

For this analysis, patients were grouped according to the type of initial pathology of mitral valve:

Group 1—patients with mitral valve stenosis and 1st or 2nd degree of mitral regurgitation

Group 2—patients with mitral valve stenosis and 3rd or 4th degree of mitral regurgitation 

Group 3—patients with isolated organic mitral valve insufficiency.

According to the type of mitral valve implant, patients were also grouped as follows:

Group 1—patients with bileaflet prostheses

Group 2—patients with disc prostheses

Group 3—patients with bioprostheses

The examination data was collected in one- and two-dimensional modes by using Philips EpiQ-7 (Koninklijke Philips N.V., The Netherlands), Philips-iE33 (Koninklijke Philips N.V., The Netherlands), Philips HDI (Koninklijke Philips N.V., The Netherlands), Siemens Acuson (Siemens Healthcare GmbH, Germany), and HP Sonos 2500 (Hewlett Packard, CA, USA) diagnostic ultrasound machines equipped with 2.5 and 3.5 MHz transthoracic sensors.

According to statistics, 80.2% of patients in Russia with acquired MVD undergo mitral valve replacement [2,5]. Thus the new ultrasonic technique was tested on patients with acquired MVD prior to and following the surgical correction. Key parameters of the heart were measured in classical echocardiographic projections such as end systolic area, end-diastolic area, end systolic volume and end diastolic volume of ventricles, ventricular ejection fraction, anatomic and geometric parameters of mitral valve, and hemodynamic features of various prostheses, atrial volume and the ratio of ventricular to atrial volumes. The patients were examined 1–2 days prior to and following the surgery-before discharge, 6 months later, 1 year later and then annually within next 5 years.

## 3. Discussion 

Organic mitral valve pathology impairs intracardiac hemodynamics predominantly in the left heart chambers [3,4,5]. First, it leads to an increase in size of the left atrium, and then changes in the geometry of the left ventricle, however less pronounced. This is due to the fact that the atrial chamber is not anatomically adapted to prolonged volumetric overload, which over time always leads to increased cavity volume, stretching of the cavity walls, decrease in atrial myocardial contractility, and onset of rhythm disturbances, which are known to negatively affect the long-term outcome of surgical correction [3,4]. Unlike the atria, the left ventricular myocardium retains its contractile functions, even if mitral hemodynamic impairment is present. Also, following the surgical correction, the geometry of the left ventricle, in contrast to the atrium, is quickly restored. Such an algorithm for remodeling of the left chambers is very characteristic of acquired MVD and leads to the volume imbalance of heart cavities mainly because of the pronounced deformation of the atria.

**Figure 1:**
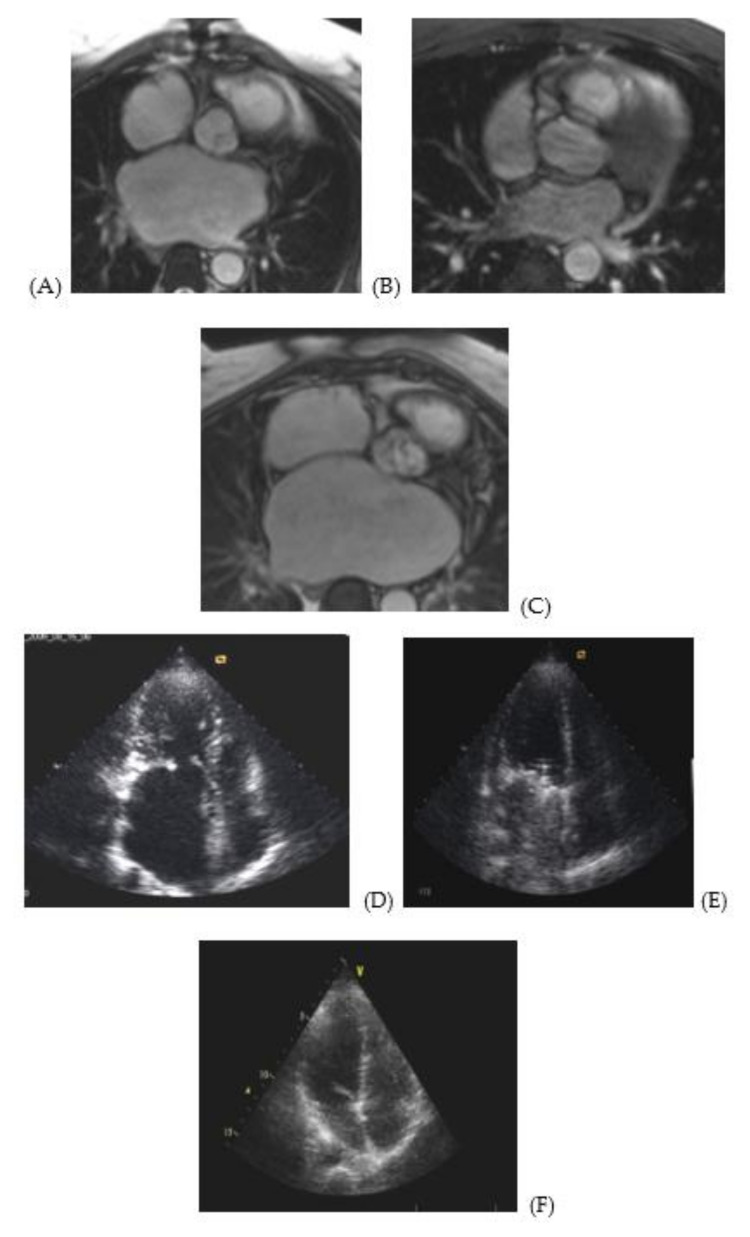
MRI and echocardiography images of atria of a 48 year-old male patient with MVD prior to surgery and 5 years later. MRI image of a cross section of the right and left atria at the level of the aortic root of a 48 year-old male patient with stenosis and mitral valve insufficiency (3d degree): (**A**) prior to surgery, (**B**) following surgery, (**C**) normal atria of a patient from the control group. Apical access echo image of the left atrium of a 48 year-old male patient with stenosis and mitral valve insufficiency (3d degree): (**D**) prior to surgery, (**E**) following surgery, (**F**) normal atria of a patient from the control group.

MRI and echocardiographic images (Figure 1) illustrate enlargement and deformation of the atria prior to surgery (Figure 1A,E) as compared to normal volume and shape (Figure 1C,F). Figure 1B,E illustrate a pronounced positive dynamics in remodeling of the atria after surgery. However, atria are not fully restored. At the same time, the left ventricle’s shape and volume are restored almost completely (Figure 1D–F).

The concept of our research is based on the fact that the imbalance of heart cavities volumes in patients with acquired MVD is an integral part of the pathogenesis of the disease.

Analysis of changes of the heart cavities volume, or pathological remodeling of heart geometry, is possible only with respect to indicators reflecting the normal ratio of the heart chambers volumes. We have encountered no information about such indicators in the literature, therefore the ratio index of cardiac cavities was determined in the control group of 20 healthy people (10 male and 10 female participants) aged 28 to 52 years. The average normal ratio index of the left heart cavities was stated at 4.0 (varying from 2.0 to 5.8), of the right heart cavities – at 1.7 (varying from 1.2 to 2.8). 

The ratio index, or the index of volume remodeling (IRV) was calculated according to the formula:

$$IRV=\frac{\mathrm{VV}\;\left(d\right)}{\mathrm{VA}\;\left(S\;\times \;L\right)}$$

VV (d)—(volume of ventricle) ventricular volume in diastole (d): the end-diastolic volume (EDV) of the right or left ventricle calculated according to the Simpson method,

VA (S × L): VA—(volume of atrium) is calculated as the product of the planimetric area of the left or right atrium (S) and the width of the corresponding cavity measured at the middle third (L).

The MRI verification of the left atrium volume figures, obtained by the proposed method, showed that the calculation error, in comparison with other methods implanted in the program of the ultrasound system, ranges from 5 to 9.3 %. The IRV deviation value in digital terms shows severity of heart geometry changes resulting from the impaired intracardiac hemodynamics; it reflects the severity of the pathological remodeling. The IRV is an ultrasound technique which allows to comprehensively evaluate the geometry of the heart as a single functional anatomical system that can respond not only to pathological factors, but also to their elimination.

## 4. Results

**Figure 2:**
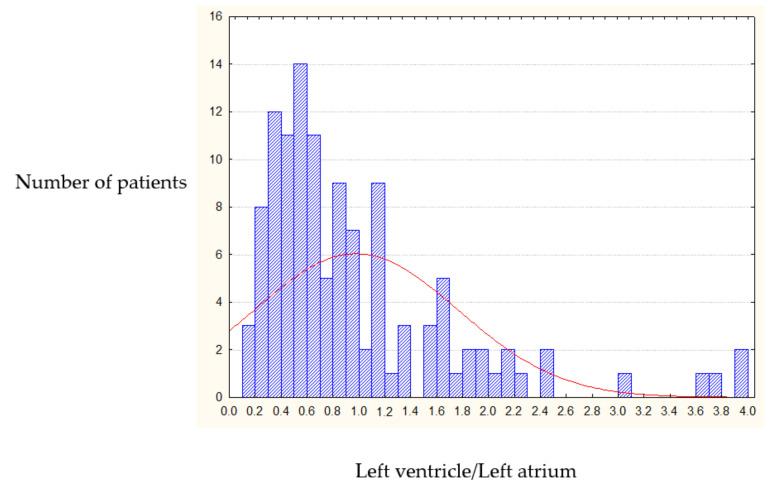
Pre-operative IRV distribution (left heart chambers).

Analysis of the IRV in 316 patients with organic MVD showed that only 3.5% of patients have normal ratio of the heart cavities volumes, a small number that can be neglected to preserve the structure of our research. The pre-operative IRV random distribution curve (conforming Rayleigh law) showed that in 96.5% of patients the index was significantly less than 2.0; and for the majority of patients the IRV was 0.6 ± 0.3 (Figure 2).

**Figure 3:**
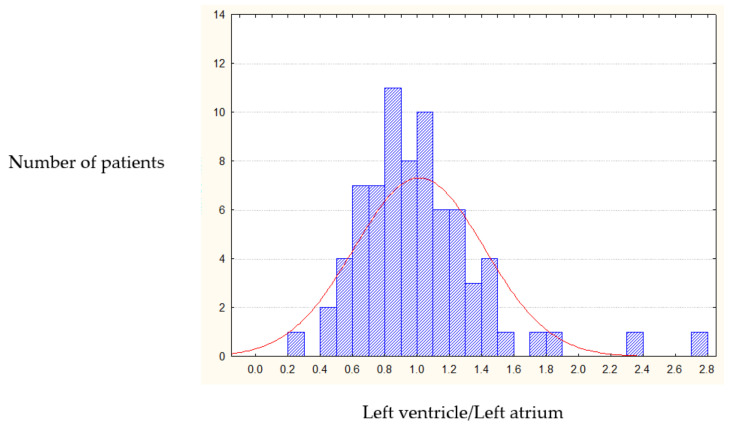
Post-operative IRV distribution (left heart chambers).

Post-operative long-term IRV dynamics (5 years after mitral valve replacement) showed that the heart cavities volume ratio returned to normal in only 8.5% of cases. Such a small percentage does not allow comparing postoperative IRV dynamics with normal ratio index. The postoperative IRV chart shows that in 54.4% of cases the IRV becomes equal to one (1.0), with the rest of data showing uniform distribution around this value (Figure 3). As a result we identified 1.0 as a threshold value for classification of post-operative heart remodeling.

Post-operative remodeling was considered as positive if the IRV increased as compared with initial value; and negative if the IRV decreased. 

**Figure 4:**
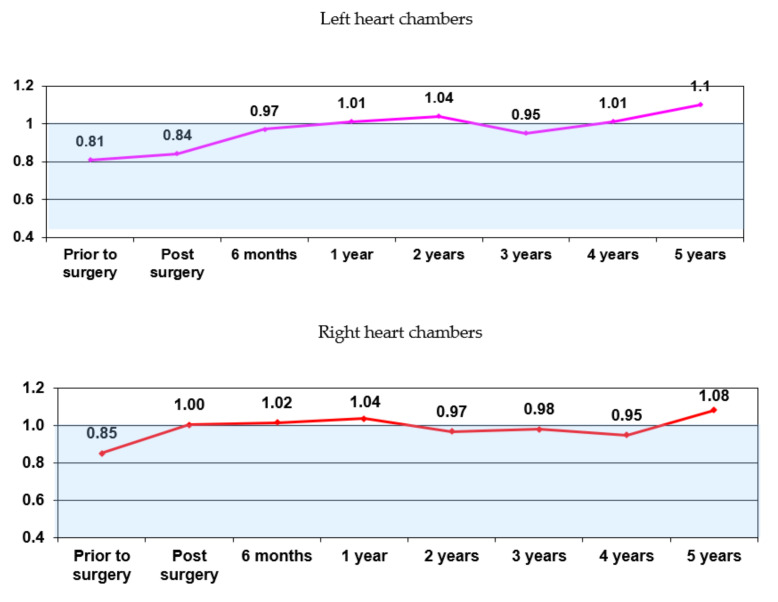
The IRV dynamics in all patients (n = 316).

Analysis of the IRV dynamics of the heart geometry restoration over the course of 5 years shows varying degree of positive remodeling in all patients; remodeling occurs in line with a single algorithm in both the left and right chambers. The chart (Figure 4) shows that the positive and negative IRV trends with respect to the threshold value (IRV = 1.0) are identical for the left and right chambers.

Obtained data indicate that the heart geometry integrally responds to changes in intracardiac hemodynamics.

**Figure 5:**
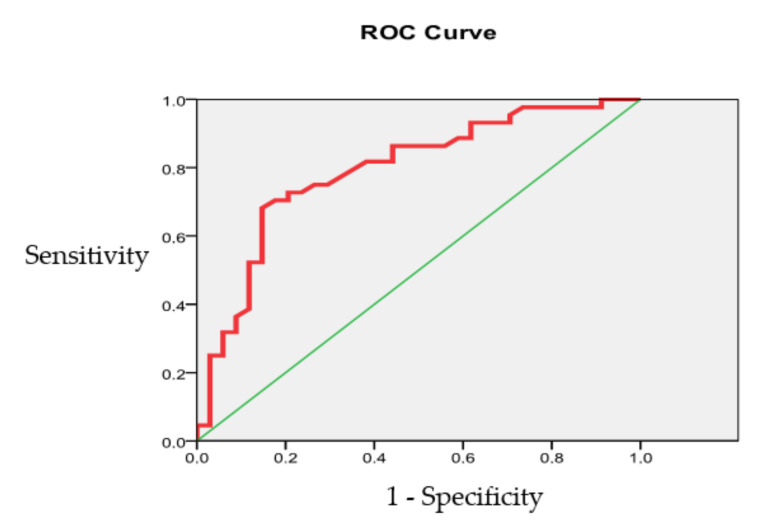
The IRV prognostic accuracy evaluation (left heart chambers).

The ROC curve analysis (Figure 5) of the IRV prognostic power based on patient data 5 years after surgery shows that the overall accuracy of the IRV model is 82.35% (CI 95%, 72.84 to 89.03).

The area under the ROC curve was 0.793 ± 0.052 (CI 95%, 0.660 to 0.871), indicating a good quality model. 

**Figure 6:**
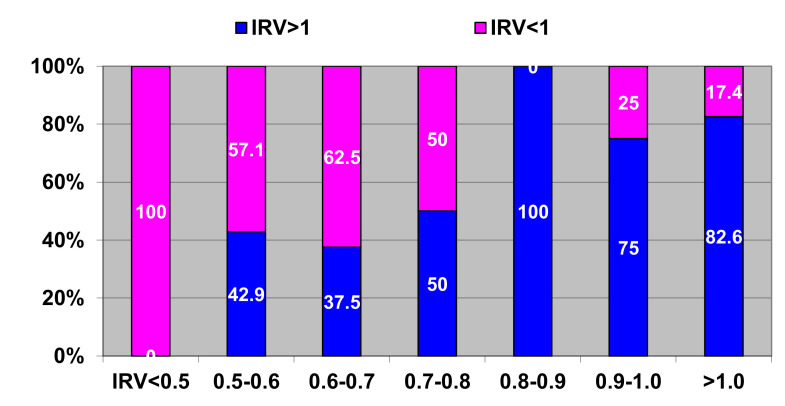
Correlations between pre-operative and long-term post-operative IRV. Horizontal axis—pre-operative IRV; vertical axis—long-term post-operative IRV with respect to threshold value. Blue color–share of post-operative IRV >1; pink color - share of post-operative IRV < 1.

Figure 6 presents correlations between initial and long-term (5 years after surgery) postoperative IRV (CI 95%: sensitivity-90.36 to 100.0, specificity-53.83 to 83.17, accuracy-74.72 to 92.41).

According to the obtained data and analysis we identified three scenarios of long-term post-operative remodeling:

Scenario 1: The IRV is restored to ≥1 (Figure 6—100% blue bar)

Scenario 2: The IRV remained unchanged at ≤0.5 despite surgical correction (Figure 6—100% pink bar)

Scenario 3: The IRV in some patients shows positive change (IRV ≥0.8) (Figure 6—blue color on two-color bars) as compared to initial values (IRV = 0.5–0.8); while in other patients the IRV is restored between ≥0.5 and ≤0.8 (Figure 6— pink color at two-color bars).

On the two bars on the right hand side we observe regression of the IRV as compared to the initial values (0.9 ≥ 1.0). The cause of this negative remodeling is mostly post-operative complications and rhythm disturbances, especially atrial fibrillation, which is a predictor of worsening post-operative prognosis [3,4]. Post-operative complications and rhythm disturbances affecting the heart geometry are a serious problem that requires a separate in-depth study and is beyond the scope of this study. 

The above-presented scenarios formed the basis of the original prognostic classification of types of pathological remodeling:

Type I (mild remodeling)— favorable prognosis (IRV ≥ 0.8–2.0); see Figure S1 in Supplementary materials as an example.

Type II (moderate remodeling)—moderately favorable prognosis (IRV 0.8–0.5); see Figure S2.

Type III (severe remodeling)—poor prognosis (IRV ≤ 0.5); see Figure S3.

Type III includes patients with the most severe, chronic pathological heart remodeling. Surgical correction of MVD contributed to a certain improvement of the intracardiac hemodynamics and clinical condition of these patients. However, cardiac resistance, especially of atria, did not allow heart geometry to restore significantly. The IRV in such patients remained at ≤0.5 even 5 years later after the surgery.

This classification is averaged and does not exclude upward or downward IRV dynamics in each scenario, depending on the effect of concomitant diseases and rhythm disturbances, which require a separate study, especially in the long term after the surgery. However, the offset variation in each scenario is limited within the range of 17–25%.

**Figure 7:**
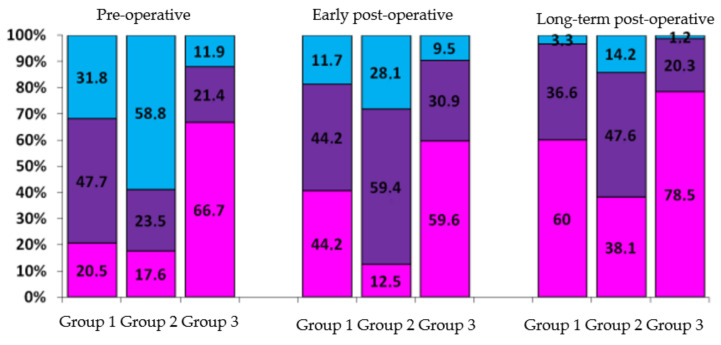
Analysis of heart geometry restoration according to types of initial pathology remodeling (in %). Group 1—patients with mitral valve stenosis and 1st or 2nd degree of mitral regurgitation. Group 2—patients with mitral valve stenosis and 3rd or 4th degree of mitral regurgitation. Group 3—patients with isolated organic mitral valve insufficiency. Pink color: share of patients with Type I remodeling; purple color: share of patients with Type II remodeling; blue color: share of patients with Type III remodeling.

An analysis of the IRV values depending on the nature and severity of the initial pathology showed that the greatest prospect for heart geometry restoration is observed in patients with isolated organic mitral valve insufficiency (Group 3, mentioned above at page 2) and patients with mitral valve stenosis and 1st or 2nd degree of mitral regurgitation (Group 1). The largest share of Type I remodeling was observed in patients of these groups; it almost doubled within 5 years following the surgery (Figure 7).

Type III remodeling was initially observed in 58.8% of patients from Group 2. The share of this remodeling type decreased to 14.2% (down 44.6%) 5 years after surgery, however it remained the biggest as compared to Groups 1 (3.3%) and 3 (1.2%); this illustrates resistance of heart chambers, especially of left atria, in the process of restoration.

We conclude that surgical interventions performed at stages of Type I and II pathological remodeling have the most favorable prognosis. At these stages it is still possible to restore the geometry of the heart chambers to normal or to Type I in more than 70% of cases.

The remodeling classification was used in the retrospective analysis of the post-operative IRV dynamics (over the course of 5 years) in order to estimate the effect of hemodynamic features of various types of prostheses on the heart chambers geometry.

**Figure 8:**
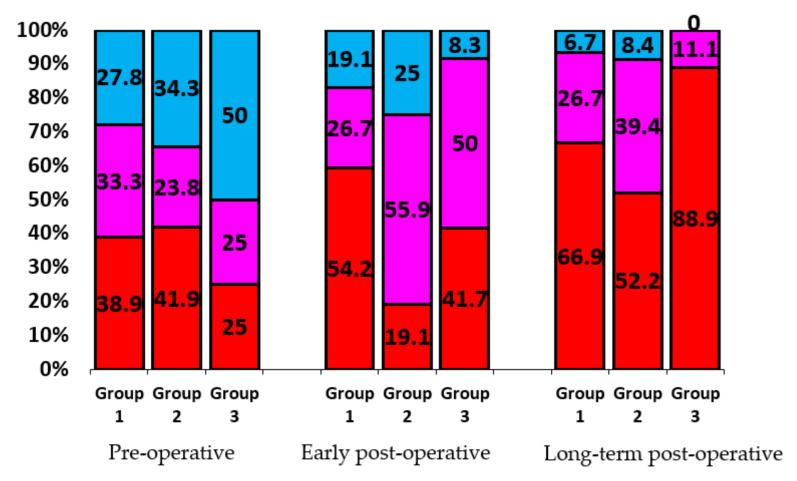
Heart remodeling versus type of mitral prosthesis (in %). Group 1 includes patients with bileaflet prostheses, Group 2—disc prostheses, Group 3—biological prostheses. Share of patients with Type I remodeling is indicated in red, Type II—pink, Type 3—blue.

The analysis of correlations between the IRV and hemodynamic features of different artificial heart valves showed that design of prosthesis has certain influence on the quality and speed of left heart chambers geometry restoration (see Figure 8).

The patients of the study group had a valve replacement surgery with the following types of implants:

disc (MIKS)

bileaflet (MedEng, ON-X, ATS, St. Jude, Carbomedics)

biological (BioLab, Sorin-Pericarbon, Carpantier-Edwards).

The best results in restoring the geometry of the heart were found in patients of Group 3 (biological prostheses). This is due to the design of these prostheses, ensuring a laminar, almost native blood flow. The design of mechanical prostheses with locking elements, crossing the passage opening, disrupts the laminar blood flow. In bileaflet prostheses the through-flow is turbulent, but central, while in disc prostheses it is turbulent and eccentric. The study showed that such a structuring of the through-flow required a period of adaptation in Group 2. In the early postoperative period, adaptation led to almost a two-fold decrease in Type I remodeling in this group (from 41.9% to 19.1%). However, the distribution of remodeling types in Group 1 (bileaflet prostheses) and Group 2 (disc prostheses) leveled out following adaptation of heart chambers to hemodynamics of disc prostheses in the long-term post-operative period. 

## 5. Conclusions

Our research offers a new ultrasound technique that can be used for a comprehensive assessment of the geometry of the heart as an integrated functional anatomical system. The results of this study are presented in the classification of patterns of heart remodeling identified in the course of the research. The IRV-based analysis of the correlations between the initial mitral valve pathology and type of prosthesis showed that the IRV has a pronounced prognostic power.

The IRV of heart chambers provides an instrument for presenting the degree of cardiac geometry deviation in digital view. As a result the information on the dynamics of remodeling of structural and geometric parameters of the heart becomes universal for monitoring the patient’s postoperative state of health by any clinicians. 

We suggest that the IRV method, as an ultrasound technique, allows us to rate the severity of pathological remodeling and should assist surgeons in determining the optimal timing for surgery as well as predicting the long-term outcome. 

This research was not aimed to study the correlation between the IRV and rhythm disturbances or other comorbid conditions. These aspects require a separate large-scale study, which gives us an interesting direction for future research.

Finally, we shall note that the pathological remodeling of heart occurs not only in case of MVD, but also in other valvular and cardiac pathologies. In these cases the IRV technique may also be used in researching remodeling patterns, providing a fresh look at the traditional approaches to determining the timing and type of treatment, including surgical, and predicting its long-term outcome. 

## 6. Patents

The IRV method has been included into the main echocardiographic protocol at the A.N. Bakulev National Medical Research Center of Cardiovascular Surgery, Moscow, since 2017. The IRV method is patented as of 15 August 2018 (Patent number 2664155).

## Supplementary Materials

The following are available online at https://www.mdpi.com/2308-3425/7/2/14/s1, Figure S1: Type I remodeling, Figure S2: Type II remodeling, Figure 3: Type III remodeling.
