# Supplementary material for: New Technique in Assessment of Heart Chambers Remodeling in Acquired Mitral Valve Defects
Source: J Cardiovasc Dev Dis. 2020 Apr 21;7(2):14. doi: 10.3390/jcdd7020014 (PMC7345903; doi:10.3390/jcdd7020014)
Supplement: Supplementary file 1 [file jcdd-07-00014-s001.pdf]

Supplementary Information

New Technique in Assessment of Heart Chambers Remodeling in Acquired Mitral Valve Defects

Leo Bokeria, Vladimir Makarenko and Tatiana Kosareva *

A.N. Bakulev National Medical Research Center of Cardiovascular Surgery, Moscow, 121552, Russia; info@bakulev.ru (L.B.); vnmakarenko@heart-house.ru (V.M.)

* Correspondence: tikosareva@bakulev.ru; Tel.: +7-910-424-57-87

Received: 5 February 2020; Accepted: 14 April 2020; Published: date

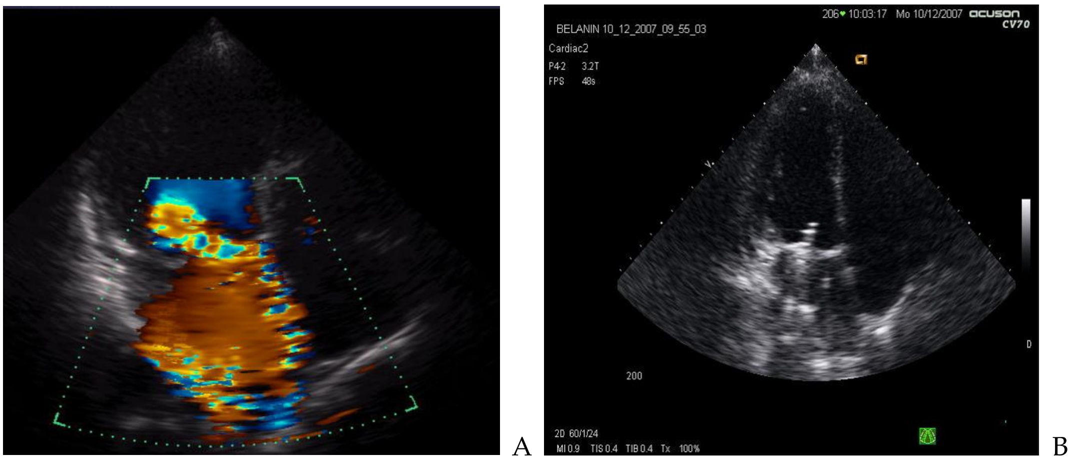

Figure S1. Type I remodeling (mild) - favorable prognosis (IRV 0.8-2.0). Patient with acute infectious endocarditis and 3d degree regurgitation: pre-operative IRV - 0.8 (Figure S1A); post-operative IRV - 1.4 (Figure S1B).

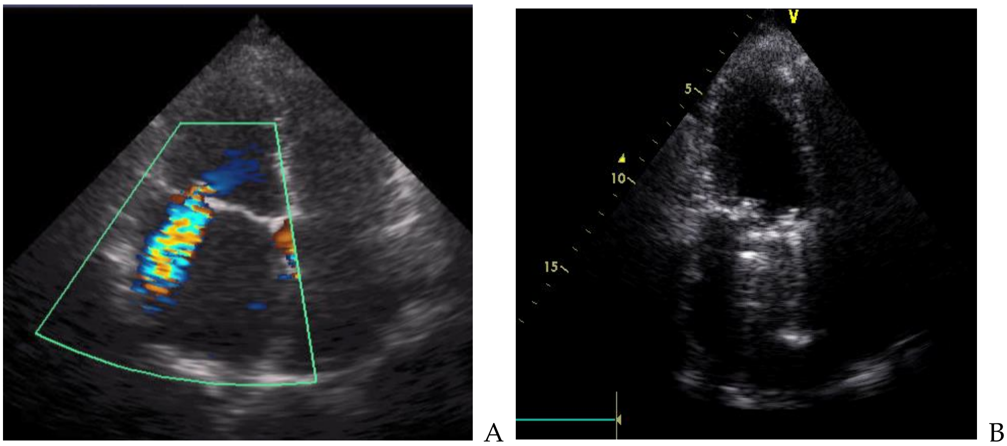

Figure S2. Type II remodeling (moderate) - moderately favorable prognosis (IRV 0.8-0.5). Patient with mitral valve stenosis and 2d degree regurgitation: pre-operative IRV – 0.6 (Figure S2A); post-operative IRV - 0.9 (Figure S2B).

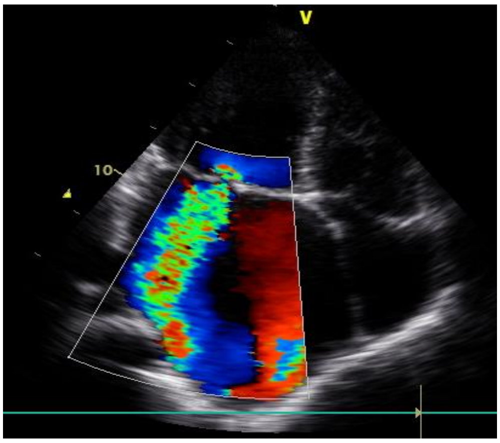
A

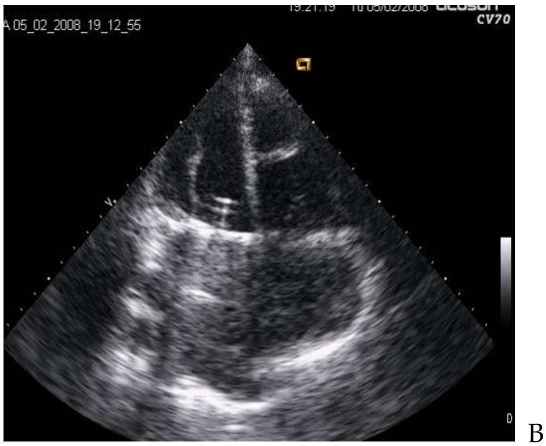
B

Figure S3. Type III remodeling (severe) - poor prognosis (IRV<0.5). Patient with mitral valve stenosis and 3d regurgitation: pre-operative IRV - 0.3 (Figure S3A); post-operative IRV - 0.5 (Figure S3B).
